# Behavioural Monitoring Underlines Habituation to Repeated Stressor Stimuli in Farmed Gilthead Sea Bream (*Sparus aurata*) Reared at a High Stocking Density

**DOI:** 10.3390/biology13110879

**Published:** 2024-10-29

**Authors:** Paul G. Holhorea, Fernando Naya-Català, Ricardo Domingo-Bretón, Federico Moroni, Álvaro Belenguer, Josep À. Calduch-Giner, Jaume Pérez-Sánchez

**Affiliations:** Nutrigenomics and Fish Growth Endocrinology Group, Institute of Aquaculture Torre de la Sal (IATS, CSIC), 12595 Castellón, Spain; paul.holhorea@csic.es (P.G.H.); fernando.naya@iats.csic.es (F.N.-C.); ricardo.domingo@csic.es (R.D.-B.); federico.moroni@csic.es (F.M.); a.belenguer@csic.es (Á.B.); j.calduch@csic.es (J.À.C.-G.)

**Keywords:** gilthead sea bream, European sea bass, stocking density, O_2_ availability, habituation, adaptation, behaviour, muscle growth

## Abstract

A confinement stress test with 75% tank space reduction and behavioural monitoring through tri-axial accelerometers externally attached to the operculum was designed. This procedure was validated by demonstrating the less pronounced stress response in gilthead sea bream than in European sea bass (950–1200 g). Our study aimed to assess habituation to high stocking densities with such procedure in gilthead sea bream. Animals (420–450 g) were reared (June–August) in a flow-through system at two stocking densities (CTRL: 10–15 kg/m^3^; HD: 18–24 kg/m^3^), with natural photoperiod and temperature (21–29 °C), and oxygen levels at 5.2–4.2 (CTRL) and 4.2–3.2 ppm (HD). At the end, blood and muscle were sampled for haematology and transcriptomic analyses, and external tissue damage was assessed by image-based scoring. Four days later, fish underwent a 45 min confinement stress test over two consecutive days. HD fish showed reduced feed intake, growth rates and haematopoietic activity. Muscle transcriptome changes indicated a shift from systemic to local growth regulation and a primed muscle regeneration over protein accretion in HD animals with slight external injuries. After stress testing, HD fish exhibited a decreased recovery time in activity and respiration rates, which was shorter after a second stressor exposure, confirming habituation to high densities.

## Simple Summary

Habituation is a strategy that involves several physiological and behavioural response mechanisms to make individuals less reactive to recurrent stimuli and cope with the challenges posed by a stressor. In order to better understand fish stress resilience and adaptive capability, it is important to assess adaptation and habituation to main aquaculture stressors, such as high stocking densities. With that purpose, in this study, a confinement stress test was designed and validated by evaluating stress response in gilthead sea bream and European sea bass. This test was further employed to explore behavioural parameters in gilthead sea bream with a different background in stocking density. Although animals reared at higher densities exhibited impaired growth performance, they also displayed physiological and behavioural adaptation strategies to partly overcome the negative effects of high-density environments. Interestingly, behavioural monitoring evidenced signs of habituation to high-density conditions in the same group of animals. Thus, evaluating habituation can help to ensure that fish are within acceptable welfare standards while optimizing industry profitability.

## 1. Introduction

Since the onset of the Blue Revolution during the mid-20th century, aquaculture production has been continuously growing at the global level [1]. Meanwhile, climate change has escalated, resulting in annual temperature increases and extreme weather events [2]. Taken together, these two processes represent a major challenge for fish farming because the intensification of production coupled with warmer temperatures increase the risk of health issues and welfare impairments [3,4,5,6,7,8]. Therefore, important research efforts over the past two decades have been directed toward understanding how stress affects the economic sustainability of fish farming by directly reducing productive and reproductive performance, but also by altering public concerns about aquaculture practices [9]. Focusing on stress dynamics, the fish stress response is initiated and controlled by corticosteroids and catecholamines that act as the primary mediators of the stress response, increasing the availability of metabolic fuels for energy production [10]. This serves to orchestrate changes in a series of secondary stress outcomes (secondary stress response), which includes changes in a wide range of physiological processes, including oxidative status, immune function, and behaviour. All this serves to encompass a freezing behaviour during which the animal can remain motionless or in a fight-or-flight state as an active response to rid itself of the threat imposed by the stressor [11,12,13]. Ultimately, this can generate a tertiary stress response that covers permanent changes in behaviour, disease resistance, growth, and reproductive performance [12]. However, contrary to common perception, not all stress is inherently negative, because moderate stress (eustress) can stimulate adaptive responses that result in an enhanced organismal resilience [14,15]. Certainly, early-life experiences of mild hypoxia may prove beneficial for gilthead sea bream, increasing survival rates by 10% and preventing growth impairment when fish are exposed to the same stimulus later in life [16]. Likewise, mild hypoxia adaptation in juvenile fish is capable of re-programming the transcriptome of skeletal muscle, improving exercise performance in Loligo system swimming tests [17]. Conversely, initially adaptive responses can become maladaptive when stressors persist or intensify beyond an organism’s coping capacity (distress), undermining health, welfare and performance [18,19]. In this regard, it is crucial to underscore how habituation can make individuals less reactive to familiar stressors and mitigate the impact of recurring challenges on their well-being [20]. This makes essential to assess the stress response before and after stressor exposure to determine how effectively farmed fish regain allostasis when faced with repetitive noxious stimuli [21].

Measurements of circulating cortisol levels are widely utilised as a stress biomarker, but their reliability in an aquatic scenario is limited by their large variability and dramatic increases by sampling itself [22,23]. Alternatively, measures of external fish appearance, haematological parameters, or tissue-specific expression patterns of epigenetically regulated genes offer the possibility of an integrated omics layer that can be reinforced by behavioural monitoring [24,25,26]. Thus, fish welfare assessment through behavioural monitoring has gained increased attention [27,28,29,30], making relevant the existence of different fish stress coping styles with variable individual and social response when facing acute and persistent stressors [31,32]. In this regard, an important stress factor in intensive aquaculture is fish stocking density [8,33]. However, in a valuable Mediterranean species like gilthead sea bream (*Sparus aurata*), despite the potential negative effects of high rearing densities on growth performance and stress indicators such as glucose and cortisol [25], it has been pointed out that the rise in stocking densities has enhanced schooling behaviour [34] and displayed swimming activity synchronised with feeding time as the main zeitgeber factor [25]. These adaptive features occurred in coincidence with changes in liver and muscle gene expression signatures of growth, antioxidant defence, and lipid metabolism, which also served to interconnect changes in skin microbiota composition with a reactive or proactive stress behaviour at high stocking densities with limited O_2_ availability [35].

According to the above findings, the repertoire of stress adaptive features is quite large and, importantly, it becomes species-specific. In addition, the potential for habituation varies not only with the species, but also with the nature and severity of the stressor [36]. Indeed, short-term exposure to repeated chasing did not induce habituation in the Senegalese sole (*Solea senegalensis*) [37], while Atlantic salmon (*Salmo salar*) seemed to be rapidly habituated to this type of stressor [38]. In the same way, exposure of yellow perch (*Perca flavescens*) to high stocking densities reduced their cortisol response after acute stressor stimulus [39], which suggests that exposure to high rearing densities could potentially confer adaptive advantages to cope with subsequent stressors. Conversely, Di Marco et al. [40] highlighted that high stocking densities in European sea bass (*Dicentrarchus labrax*) did not enhance the ability of this marine fish to cope with additional stressors, which suggests that this species is not likely to adapt or habituate to common stressful environmental conditions [41]. However, it remains unclear how the habituation of farmed fish to the main aquaculture stressors can be routinely assessed, and more importantly, how the measured stress susceptibility can be altered by a different life background. Thus, the primary aim of the present study was the individual tracking in gilthead sea bream of behavioural parameters (swimming activity and respiratory frequency) as indicators of habituation to high stocking densities, using tri-axial accelerometers externally attached to the operculum (AEFishBIT data-loggers; [28]). As part of the experimental setup, gilthead sea bream and European sea bass, species showing a very different stress-responsiveness [25,36], were used to check the accuracy of our behavioural approach to assess differences in stress dynamics and responsiveness following a single/repetitive confinement stress test. Such approach was complemented by measurements of growth performance, external tissue damage, haematology, and skeletal muscle transcriptomics in gilthead sea bream.

## 2. Materials and Methods

### 2.1. Ethics Statement

All procedures were approved by the Ethics and Animal Welfare Committee of the Institute of Aquaculture Torre de la Sal (IATS), CSIC Ethics Committee (permission 1135/2021), and Generalitat Valenciana (permission 2021-VSC-PEA-0192). They were carried out in the IATS’s registered aquaculture infrastructure facility (code ES1200330001055) in accordance with the principles published in the European Animal Directive (2010/63/EU) and Spanish laws (Royal Decree RD53/2013) for the protection of animals used in scientific experiments.

### 2.2. Animals

Gilthead sea bream and European sea bass of Mediterranean origin (Avramar, Burriana, Spain) were reared from early life stages in a flow-through system (3000 L tanks) at the indoor experimental facilities of Institute of Aquaculture Torre de la Sal (IATS-CSIC, Spain) until two/three years of age. Rearing conditions followed the natural photoperiod and temperature (21 to 29 °C) at our latitude (40°5′ N; 0°10′ E). Fish were fed commercial diets (BioMar, Palencia, Spain; EFICO 3053, gilthead sea bream; EFICO 4057, European sea bass) near to visual satiety with automated feeders, 1–3 times per day, 3–7 days per week, depending on season and fish size. At the early life stages, all fish were pit-tagged in the dorsal musculature with passive integrated transponders (ID-100A 1.25 Nano Transponder; Trovan, Madrid, Spain, 7 mm length, 1.25 mm diameter) for individual tracking. Water O_2_ concentration was maintained higher than 75% saturation and unionised ammonia remained below 0.05 mg/L. Fish behaviour was routinely checked using video cameras on the top of the tank.

### 2.3. Experimental Setup and Fish Sampling

**Figure 1:**
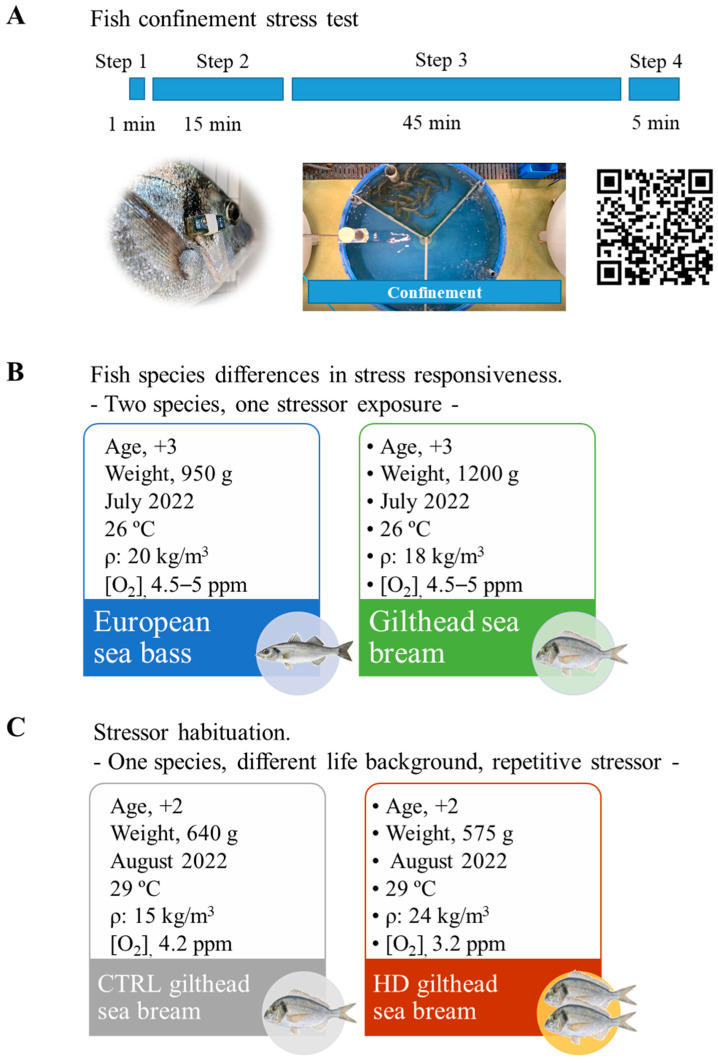
(**A**) Confinement method explained in four steps: Step 1 (structure insertion), Step 2 (folded structure), Step 3 (unfolded structure and fish confinement), Step 4 (folded structure and removal). Video of the process is available accessing this link: https://vimeo.com/1015372239 (accessed on 2 October 2024) or scanning the QR code. Information on fish and water parameters (**B**) when European sea bass and gilthead sea bream were exposed to one confinement test and (**C**) when gilthead sea bream reared at two different densities were exposed to repetitive confinement tests.

For testing the accuracy of our behavioural system, three-year-old gilthead sea bream (*n* = 12) and European sea bass (*n* = 12) (950–1200 g) individuals were anaesthetised with 0.1 g/L MS-222 (Sigma, Saint Louis, MO, USA). Fish were individually weighed and measured using a FR-200 FishReader W (Trovan), and before returning to their original 3000 L tanks, AEFishBIT devices were externally attached to the operculum for the simultaneous monitoring of physical activity and respiratory frequency before, during, and after a single stressor exposure (see Section 2.4; Figure 1A). This stress test was conducted in July 2022, using fish of the same class of age reared before testing in similar conditions of temperature (26 °C), O_2_ concentration (4.5–5 ppm), and stocking density (18–20 kg/m^3^) (Figure 1B). Subsequently, habituation to high stocking density was assessed using gilthead sea bream individuals reared at different stocking densities for over two months prior to being subjected to a repetitive stressor stimulus (Figure 1C). To establish these distinct life backgrounds, in early June 2022, two-year-old fish with an initial body weight of 434.9 ± 8.2 g (420–450 g) were allocated in duplicate 3000 L tanks at two different initial stocking densities (control density, CTRL: 65 fish, 10 kg/m^3^; high density, HD: 115 fish, 18 kg/m^3^). Over the course of two months (June–August 2022), the flow of inlet water was regulated daily to maintain differentially controlled the water O_2_ concentration (CTRL, 4–5 ppm, 70–85% saturation; HD, 3–4 ppm, 50–65% saturation). At the end of the pre-conditioning trial, 8 fish per experimental condition were anaesthetised with MS-222, and blood samples were taken from the caudal vessels with heparinised syringes to assess the haematocrit (Ht) and haemoglobin (Hb) concentrations. Muscle fat content was determined in situ with a Distell Fish Fat-meter, FM 692 (Distell Ltd., West Lothian, UK), and photographs were also taken for the evaluation of fish external damage (cataracts, exophthalmia, gill status, fin damage, and skin lesions) by using a scoring system from 1 to 5 adapted from Hoyle et al. [42], where 5 indicated maximum (although mild) damage in our study. These sampled fish were then euthanised by cervical section, liver weight was determined, and portions of dorsal white skeletal muscle (150–200 mg) were excised and collected in RNAlater (Ambion, Austin, TX, USA) for its storage at −80 °C until RNA extraction for gene expression analyses. Furthermore, image-based welfare scoring was conducted on 20 additional fish from each experimental condition (a total of 28 fish per group), which were then returned to their original tanks. Four days after this sample collection, AEFishBIT devices (20 per experimental condition) were externally attached to the operculum of anaesthetised fish (subsequently returned to their tanks), and fish from both stocking densities were exposed to a repetitive stressor as indicated below (Section 2.4).

### 2.4. Confinement Stress Test and Behavioural Recording

The confinement stress test was designed to be applied as a reproducible and single/repetitive stressor without directly handling the fish or compromising water quality (Figure 1A). Briefly, it entailed placing a folded self-made PVC confinement structure into the tank for 15 min, allowing for the fish to become acclimated to the new object. Subsequently, the structure was unfolded, and fish were confined to one portion of the tank for a fixed time, reducing the available space up to 75% for 45 min. Following confinement, the structure was folded again, maintained in the tank for 5 additional min, and then removed, enabling the fish to resume free swimming. The assessment of the behavioural response was performed using the AEFishBIT data-logger, externally attached to the operculum (one day before the stress test) for the simultaneous monitoring of physical activity and respiratory frequency. AEFishBIT is a stand-alone, small, and lightweight motion-embedded-microsystem with a tri-axial accelerometer that monitors physical activity by mapping of the accelerations in *X*- and *Y*-axes, while the operculum beats (*Z*-axis) serve as a measure of respiratory frequency [28]. The devices were programmed for data acquisition for 2 min every 15 min along two consecutive days, during which fish remained unfed. The sampling frequency of the AEFishBIT device was 100 Hz, and pre-processing of raw data was carried out with authors’ proprietary software as described elsewhere [43,44].

### 2.5. Haematological and Gene Expression Analyses

Hb was assessed using a Hemocue Hb 201+ (Hemocue, Ängelholm, Sweden). Measures of Ht were conducted using heparinised capillary tubes centrifuged at 1500× *g* for 30 min in a Sigma 1–14 centrifuge (Sigma, Osterode am Harz, Germany) and quantified using a microhematocrit reader, following the manufacturers’ instructions. Tissue RNA was extracted using the MagMAX-96 total RNA isolation kit (Life Technologies, Carlsbad, CA, USA) after tissue homogenisation in TRI reagent following manufacturers’ instructions. RNA quantity and purity was determined by Nanodrop (Thermo Scientific, Waltham, MA, USA) with absorbance ratios at 260 nm/280 nm of 1.9–2.1. Reverse transcription (RT) of 500 ng of total RNA was performed with random decamers using the High-Capacity cDNA Archive Kit (Applied Biosystems, Foster Coty, CA, USA). RT reactions were incubated for 10 min at 25 °C and 2 h at 37 °C. Negative control reactions were run without reverse transcriptase. Real-time quantitative PCR was carried out with an Eppendorf Mastercycler Ep Realplex, using 96-well PCR array layouts designed for the simultaneous profiling of 44 selected genes of white skeletal muscle (Table 1). The analysed transcripts of muscle included markers of the growth hormone and the insulin-like growth factor (GH/IGF) system (10), muscle cell growth (8), immune response (5), oxidative metabolism and energy sensing (12), and antioxidant defence (9). Specific PCR primer pair sequences are listed in Supplementary Table S1. Controls of general PCR performance were included on each array, and all the pipetting operations were performed by means of an EpMotion 5070 Liquid Handling Robot (Eppendorf, Hamburg, Germany). Briefly, reverse transcription reactions were diluted to convenient concentrations and the equivalent of 660 pg of total input RNA was used in a 25 μL volume for each PCR reaction. PCR wells contained a 2× SYBR Green Master Mix (Bio-Rad, Hercules, CA, USA) and specific primers at a final concentration of 0.9 μM were used to obtain amplicons of 50–150 bp in length. The PCR amplification program consisted of an initial denaturation step at 95 °C for 3 min, followed by 40 cycles of denaturation for 15 s at 95 °C and annealing/extension for 60 s at 60 °C. The efficiency of the PCR reactions was consistently higher than 90% and similar among all genes. The specificity of the reactions was verified by melting curve analysis (ramping rates of 0.5 °C/10 s over a temperature range of 55–95 °C) and linearity of serial dilutions of RT reactions. Gene expression was calculated using the delta–delta Ct method [45]. β-actin was tested for gene expression stability (GeNorm software, version 3.5), M score = 0.21), and it was used as housekeeping gene in the normalisation procedure. For multigene expression analysis, all values in the muscle were referenced to the expression levels of follistatin in CTRL fish with an arbitrary assigned value of 1.

### 2.6. Statistical Analysis

Statistically significant differences (*p* < 0.05) in growth performance, blood haematology, external tissue damage, gene expression, and behavioural response between the experimental groups were assessed by Student’s *t*-test, using SigmaPlot software 14.5 (Systat Software, San Jose, CA, USA).

## 3. Results

### 3.1. Species-Specific Responsiveness After One Single Stressor Stimulus

**Figure 2:**
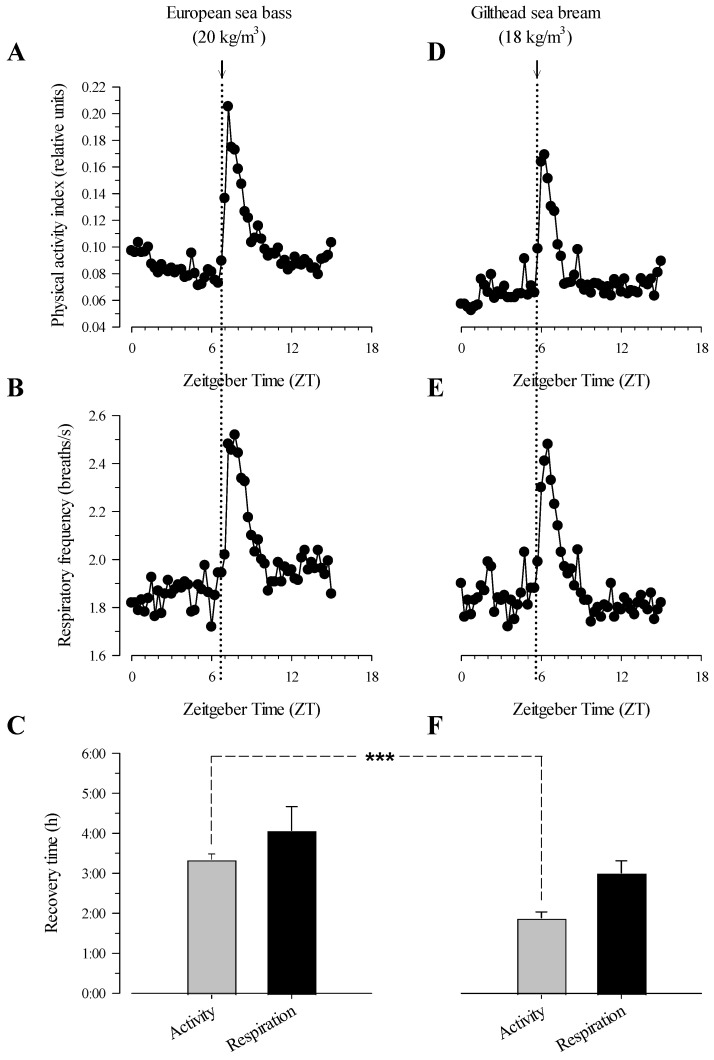
European sea bass and gilthead sea bream AEFishBIT records of physical activity (**A**,**D**) and respiratory frequency (**B**,**E**) before, during, and after the confinement test. Recovery time after the stress test of both species (**C**,**F**). Measures (black circles) were taken every 15 min. Arrows in vertical dotted lines indicate the beginning of the confinement test. *** indicates significant differences between the fish species in recovery time (*p* < 0.001). Recovery time was calculated as the required time for activity and respiration to reach values close (<15% difference) to the basal levels found before the stress test of each fish (*n* = 8).

As shown in Figure 2, AEFishBIT measurements of physical activity and respiratory frequency in three-year-old gilthead sea bream and European sea bass showed a fast and pronounced rise following a single stressor exposure. The achieved increment in physical activity was slightly higher in European sea bass (181%) than in gilthead sea bream (153%) (Figure 2A,D), while the registered increase in respiratory frequency was almost identical in both species (European sea bass, 37%; gilthead sea bream 36%) (Figure 2B,E). Likewise, the recovery time for the restoration of the basal physical activity was significantly higher (*p* < 0.001) in European sea bass (3 h) than in gilthead sea bream (2 h; Figure 2C,F). The same trend was found for the restoration of basal respiratory frequency, although differences were not enough to be statistically significant (*p* > 0.1), with overall values varying between 3 h and 4 h.

### 3.2. Effects of High Stocking Density on Growth Performance and Indicators of External Damage

Initial body weight and body length did not differ significantly (*p* > 0.05) in gilthead sea bream with a different life background (Table 2). However, by the end of the trial, the increase in body weight and body length were markedly lower in the HD group (*p* < 0.001), as a result of a significantly lower feed intake (*p* < 0.001) that rendered a 22% reduction in specific growth rates (SGR; HD: 0.47%; CTRL: 0.60%). This also led to a significant decrease (*p* < 0.001) in Fulton’s body condition factor K (CFK), which varied from 2.72 in CTRL fish to 2.62 in HD fish, along with lower muscle fat content (*p* < 0.05), which is indicative of a leaner body shape in HD fish. These findings were accompanied by a significant rise (*p* < 0.05) in the feed conversion ratio (FCR) from 1.57 in CTRL fish to 1.72 in HD fish, in concurrence with a significant reduction (*p* < 0.05) in liver weight and hepatosomatic index (HSI) as stocking density increased. Regarding blood haematological parameters, Hb concentration exhibited a significant decrease (*p* < 0.01) in HD fish (8.89 g/dL compared to CTRL fish (10.17 g/dL). Likewise, Ht levels were significantly lower (*p* < 0.001) in the HD group (38.09%) compared to those found in the CTRL group (49.16%).

**Figure 3:**
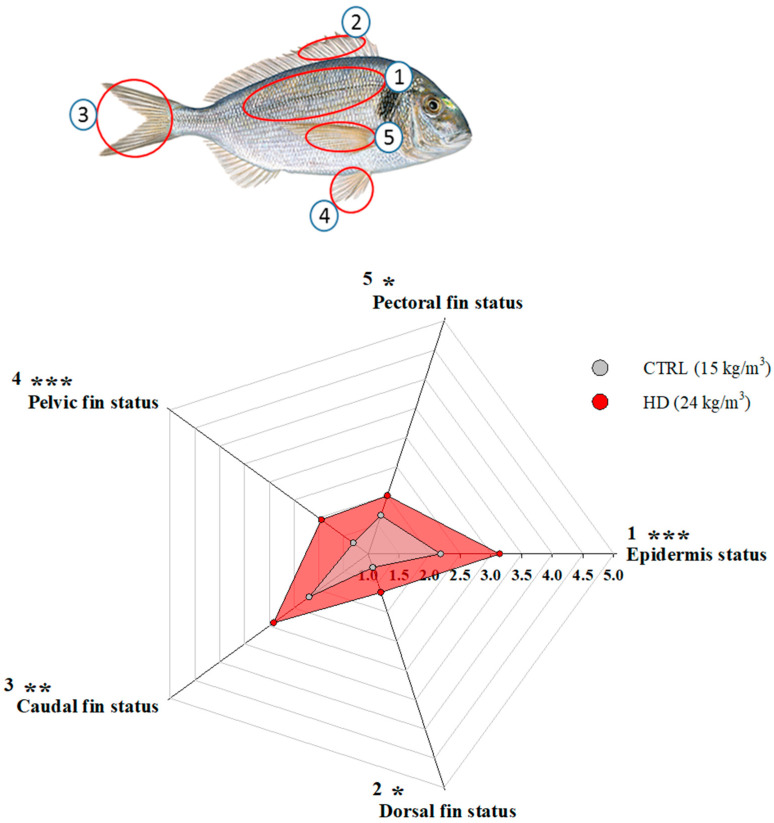
Radar plot representing external welfare indicators of gilthead sea bream reared at two different densities (scoring system from 1 to 5 adapted from Hoyle et al. [42]). Numbered fish body parts are indicated for visualisation. Coloured points are the mean (*n* = 28) of each welfare indicator. Asterisks indicate statistically significant differences (* *p* < 0.05, ** *p* < 0.01 and *** *p* < 0.001, Student’s *t*-test).

Welfare scores of external damages were also altered by the stocking density, as represented in the radar plot in Figure 3. The epidermal status was slightly, although significantly (*p* < 0.001) affected by the stocking density, with HD fish showing more signs of scale shedding, categorised as minor injuries. Dorsal, caudal, pelvic, and pectoral fin status were also significantly (*p* < 0.05) affected in the same way by the higher stocking density. Signs of cataracts or exophthalmia were absent in both fish groups, and gill status was visually similar and in good condition in all fish.

### 3.3. Changes in Muscle Gene Expression with the Increase of Stocking Densities

**Figure 4:**
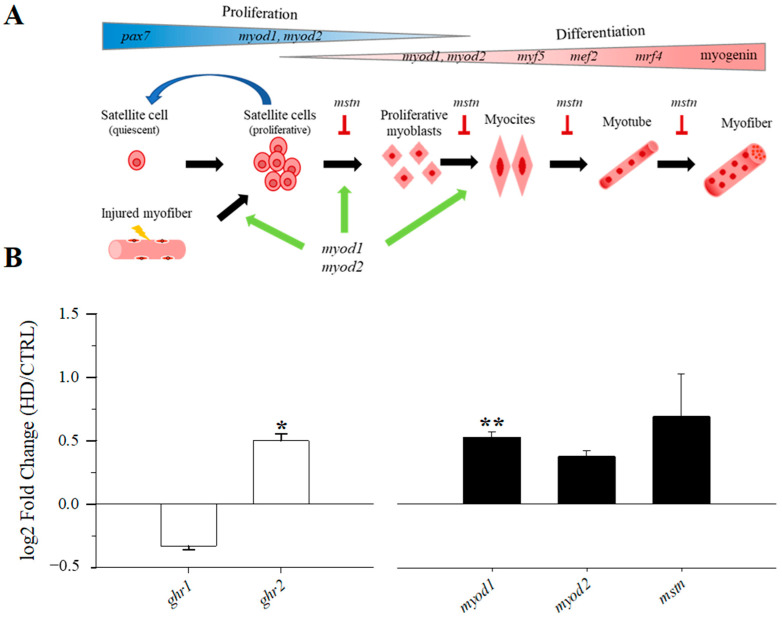
(**A**) Illustration of skeletal muscle regeneration, adapted from Romagnoli et al. [46]. Symbols of the genes involved in proliferation and differentiation processes of satellite cells are presented (top of the figure). Green sharp arrows indicate stimulation (*myod1*, *myod2*) and red blunt arrows refer to inhibition (*mstn*). (**B**) Log2 fold changes in relative expression values of white skeletal muscle growth and regeneration genes (±SEM) comparing HD vs. CTRL groups. Asterisks indicate statistically significant differences (* *p* < 0.05, ** *p* < 0.01).

In gilthead sea bream reared at different stocking densities, all genes analysed in the muscle PCR array exhibited detectable expression levels. The expression profile of white skeletal muscle displayed normal relative expression levels of the different groups of examined genes, with, for example, very low relative levels in genes related to the immune response (i.e., *il1β*, *il6*, *il8*, *il10*, *il12β*). Remarkably, most genes did not present statistically significant differences (*p* > 0.1) due to the different rearing densities (Supplementary Table S2), and only two genes related to the GH/IGF system (*ghr1*, *ghr2*) and one associated with muscle cell growth (*myod1*) varied significantly (*p* < 0.05) or showed a trend to significance (*p* < 0.1; Supplementary Table S2). A graphical representation of the muscle growth and regeneration pathways is shown in Figure 4A. In HD fish, *ghr2* and *myod1* were significantly up-regulated in comparison with CTRL fish (*p* < 0.05; Figure 4B). Additionally, *myod2* and *mstn* followed a similar trend, being up-regulated in HD fish, although differences did not reach statistical significance (*p* > 0.1). Conversely, *ghr1* presented a trend (*p* < 0.1) for a down-regulation in HD fish. The observed significant up-regulation of *myod1*, together with the numerical increase in *myod2*, indicates a potential stimulation of the proliferation of satellite cells, whereas the variation in *mstn* points to a possible inhibition of the differentiation of satellite cells into muscle fibres.

### 3.4. Effective Stressor Habituation After High Stocking Rearing

**Figure 5:**
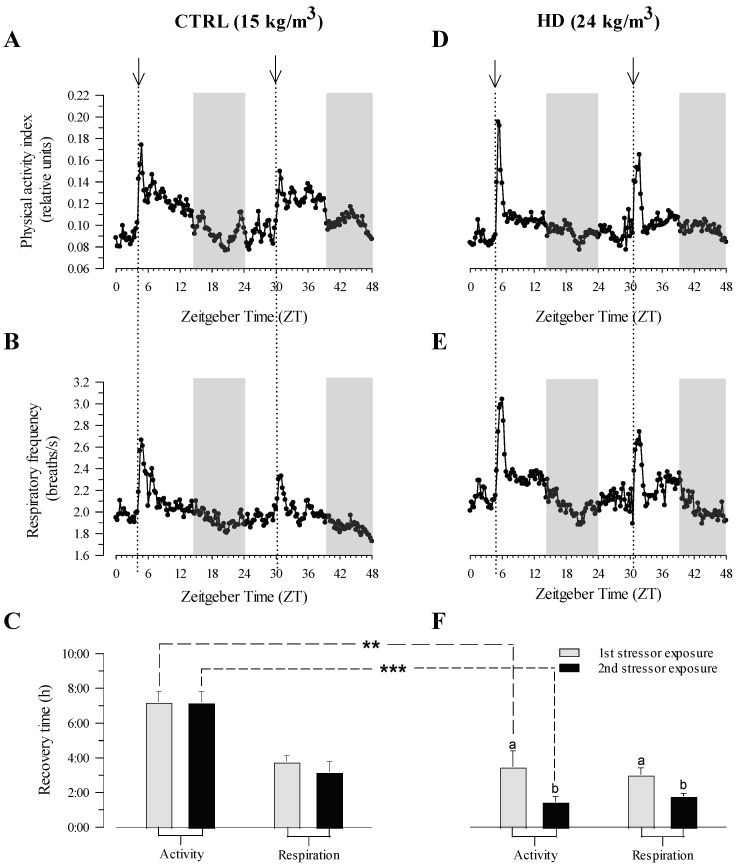
Representation of physical activity (**A**,**D**) and respiratory frequency (**B**,**E**) AEFishBIT records before, during, and after the confinement test in gilthead sea bream reared at two different stocking densities (CTRL 15 kg/m^3^; HD, 24 kg/m^3^). Recovery time after each stress test of both density groups (**C**,**F**). Measures (black circles) were taken every 15 min. Arrows in vertical dotted lines indicate the beginning of each confinement test. Asterisks indicate statistically significant differences between experimental groups in recovery time (** *p* < 0.01, *** *p* < 0.001). Different letters indicate significant differences between first and second stressor exposure (*p* < 0.05). Recovery time was calculated as the required time for activity and respiration to reach values close (<15% difference) to the basal levels found before the stress test of each fish (*n* = 7–10).

Gilthead sea bream exhibited an immediate response to the application of the stress test in terms of physical activity and respiratory frequency across both density groups (Figure 5A,B,D,E). However, the peak of the response in physical activity and respiratory frequency exhibited a smaller increase in the CTRL group compared to the HD group during both stressor exposures. Furthermore, following the initial stressor exposure, HD fish were able to return to their baseline levels of activity and respiration more swiftly than CTRL fish, particularly in terms of activity, where the difference in recovery time was significant (*p* < 0.01) (Figure 5C,F). This pattern persisted with the second stressor exposure, with both activity (*p* < 0.001) and respiration recovering faster in the HD group. Additionally, while there was no notable distinction in recovery time between the first and second stressor exposure in the CTRL group (Figure 5C), a shorter recovery time (*p* < 0.05) was observed in the HD group after the second stressor exposure in both activity and respiration (Figure 5F).

## 4. Discussion

In light of the growing demand for fish supply and the impact of rising ocean temperatures, understanding fish stress has become essential in daily aquaculture operations [33,47]. This is partly due to the increasing impact of the main aquaculture stressors, such as low O_2_ availability, increased stocking densities, and high temperature [8,48,49]. Moreover, the diversity of stress coping styles observed across species and even within populations makes necessary to develop new stress assessment techniques [50]. Certainly, it is important to examine to what extent animals are able to adapt or habituate to episodes of multiple stressors, such as high temperatures or stocking densities, which are likely to affect water quality through reduced O_2_ availability [51]. Thus, in this study, attention was focused on behavioural traits as main indicators of physiological habituation to the environment. For that purpose, a confinement stress test as single or recurrent stressor stimulus was first designed and validated, with special emphasis on the different time course of stress response in gilthead sea bream and European sea bass. Subsequently, individuals of gilthead sea bream were kept at different stocking densities to examine the effect of stocking density pre-conditioning on the achieved stress response. This allowed to disclose that gilthead sea bream pre-adapted to high rearing densities recuperated faster after confinement exposure. Furthermore, recovery times in these animals were even lower after the application of a second stressor stimulus, which reinforced the notion of a certain habituation of HD fish to a high stocking density.

Taking a closer look, both European sea bass and gilthead sea bream exhibited a strong behavioural alarm following the start of the confinement test. However, European sea bass showed a greater increase in physical activity (Figure 2A,D), while variations in respiratory frequency were almost similar in both species (Figure 2B,E). These observations were consistent with the natural behaviour and physiological traits of European sea bass as a fast-swimming predator, with operculum and body–tail movements allowing for its explosive movements, which would be supported mostly by anaerobic white muscle fibres [44,52,53]. Certainly, gilthead sea bream typically demonstrates strong social cohesion by forming schools, while European sea bass are solitary and do not engage in group living behaviours in the wild [54]. In this line, video-recording of farmed European sea bass often fails to reveal pre-feeding activity with a clear shift from a slow and erratic swimming pattern to an increasingly organised schooling behaviour. However, the individual behavioural tracking with the AEFishBIT data-logger clearly evidenced that two-year old European sea bass (650 g mean body weight) had an anticipatory feeding response [44], albeit less intense and visibly organised than in gilthead sea bream. Indeed, pre-feeding activity of gilthead sea bream becomes clearly evident through both video recordings and AEFishBIT measures, characterised by a marked transition to more organised schooling behaviour and increased swimming activity as feeding time approaches [16,28,55]. Hence, the solitary and predatory nature of European sea bass makes this farmed fish more reactive and less habituated to group settings or confined conditions compared to gilthead sea bream. This notion is supported by the study of Samaras et al. [56], which highlighted an enhanced impact of different chronic stress regimes on growth performance and plasma cortisol levels in European sea bass. Certainly, Fanouraki et al. [36] also found an enhanced cortisol response of European sea bass, with a longer recovery time compared to other Mediterranean farmed fish. This observation was further supported in this study by a longer recovery time of physical activity (Figure 2C,F), following stress confinement, in European sea bass (3–5 h) than in gilthead sea bream (2–3 h). Therefore, the integration of the confinement test alongside individual accelerometer tracking is proved to be a robust method to precisely evaluate stress condition and susceptibility in farmed fish, and Mediterranean fish in particular.

Gilthead sea bream adaptations to high stocking densities have been extensively documented in previous studies, revealing a wide range of both metabolic [57,58,59,60] and behavioural changes [25,32,34]. Accordingly, it was found herein that fish stocked at a high density (24 kg/m^3^, 50–65% O_2_ saturation) experienced a reduced feed intake, leading to a decrease in growth performance and muscle fat content that was concurrent with an impaired feed conversion compared to fish reared under standard conditions (15 kg/m^3^, 70–85% O_2_ saturation; Table 2). The adverse effect of high stocking densities on fish growth and feed intake has been largely documented, although the influence on feed conversion has long remained controversial [3], being highly dependent on species tolerance to confinement stress, experimental conditions, and fish age and size. Indeed, a recent meta-analysis with 42 fish species from 52 relevant experiences highlighted a high FCR variability with no conclusive impact (positive or negative) of high stocking densities on nutrient utilisation [61]. In our case, the observed physiological changes were accompanied by reduced Ht and Hb (Table 2), a scenario that reduces O_2_ carrying capacity in HD fish. This suggests that HD fish might be in a hypo-metabolic state. Certainly, under conditions of hypoxia or mild hypoxia, fish are able to reduce energy consumption through a diminished basal metabolism [62,63]. In addition, fish reared at high stocking densities also undergo energy redistribution, prioritizing activities essential for survival overgrowth [25,64]. This adaptive energy allocation helps to maintain a high level of swimming activity, which is considered crucial in crowded environments where competition for space and resources is perceived as high by individuals [8]. Consequently, the energy that would normally support growth is instead diverted to sustaining physical activity and other critical functions, leading to the observed reductions in growth performance. Moreover, under hypoxic conditions, large amounts of reactive oxygen species (ROS) may be produced in mitochondria [63], which can lead to oxidative stress. In order to mitigate this enhanced risk, fish respond by actively reducing feed intake [65], as part of an oxystatic control mechanism that limits the metabolic use of O_2_ [66]. In any case, the social dynamics inherent to the schooling behaviour of fish, and of gilthead sea bream in particular, affect how individuals co-exist and compete with each other [67,68,69,70]. In this regard, it must be noted that no aggressive behaviour was observed in HD fish in the present study. Therefore, as previously stated [25,32,71], the occurrence of slight signs of external damage in not extremely high stocking densities would be due to involuntary collisions during feeding time rather than direct aggressions among individuals. The absence of an up-regulated expression pattern of any of the pro-inflammatory cytokines (*il1β*, *il6*, *il8*, and *il12*), included in our customised muscle PCR array, provides indirect evidence of this assumption (Supplementary Table S2). Indeed, severe external damage associated with aggressive behaviour or mechanically produced injuries (vertically inserted needle into the dorsal musculature) seems to trigger a pronounced up-regulation of *il6* and *il15* in gilthead sea bream [72].

In contrast to the immune regulatory markers, expression of genes related to the endocrine growth cascade, particularly *ghr1* and *ghr2*, was differently expressed in the white skeletal muscle of CTRL and HD fish (Figure 4). Certainly, Holhorea et al. [25] recently highlighted the opposite regulation of growth hormone receptors by stocking density in liver and white skeletal muscle. Accordingly, high stocking density down-regulated the expression of hepatic *ghr1* in concurrence with the up-regulation of *ghr2* in skeletal muscle. This endocrine readjustment would be part of the growth regulatory mechanisms that promote a shift from systemic to local muscle growth regulation in a challenging environment [73]. This finding was corroborated herein by a clear up-regulation of muscle *ghr2* in HD fish, which was concomitant to some extent with a down-regulated expression of *ghr1*. This result reinforced a decreased muscle *ghr1*/*ghr2* expression ratio in our experimental setup. This differential gene regulation was extensive to myogenic genes, particularly *myod1*, a key regulator of myogenesis that orchestrates both muscle cell proliferation and differentiation [74,75,76]. However, the up-regulation of *myod1* was concurrent with the enhanced expression of *mstn*, a potent inhibitor of muscle growth that suppresses the activation, proliferation, and differentiation of satellite cells into mature muscle fibres [77,78,79]. Consequently, this regulatory interplay suggests that the processes of proliferation and differentiation of skeletal muscle in HD fish are directed towards muscle fibre regeneration rather than net muscle protein accretion. This would be adaptive in nature in an environment with a high risk of involuntary collisions leading to minor skin erosion and epidermal/muscle injuries.

Ultimately, adaptation to high stocking densities encourages behavioural plasticity [80,81] as part of the different habituation strategies to a challenging condition [12,82]. However, increasing experimental evidence points to complex cognitive abilities and behaviours of fish [83,84], which may differ depending on the type and intensity of stress [85,86] and, more importantly, on fish species. Notably, this enables some fish species to become habituated [20,38,87,88] or not [37,89,90] to specific stress stimuli. This reinforces the interest in applying behavioural approaches to improve both production and welfare in aquaculture practice [91]. In that sense, a shorter recovery time of physical activity following the application of the stress test indicated that HD gilthead sea bream displayed herein a certain degree of habituation to high stocking densities. This observation is consistent with the idea that physical activity is a good proxy of a changing behavioural condition in fish [12]. In contrast, respiration appears to be less sensitive to confinement stress or more quickly regulated by intrinsic physiological mechanisms when considering the magnitude of change, but not necessarily when comparing the recovery time between separate tests (single vs. repetitive stress test; Figure 2 and Figure 5). In any case, following repeated stressor exposure, HD fish showed a shorter recovery time in both physical and respiration activity, clearly indicating a more efficient stress response through a prior habituation to a high stocking density. In a practical sense, such habituation measurements can serve as an effective tool to assess the degree of adaptation of farmed fish to intensive culture systems. Additionally, it would also contribute to establishing the maximum culture density for a given class of size, temperature, and O_2_ availability. In that sense, if fish show signs of habituation, it is likely that the stocking density is within acceptable limits. Conversely, a lack of habituation may reveal that the current density exceeds the fish’s tolerance. In the latter case, adjustments in density, temperature, and/or O_2_ concentration would be necessary to find the right balance between aquaculture profitability and farmed fish welfare in a context of climate change and co-existence of land- and sea-based farms, where aquaculture stressors interact synergistically [25,51,71].

## 5. Conclusions

Key findings demonstrated that gilthead sea bream exhibited a less pronounced and prolonged behavioural stress response compared to European sea bass, consistent with their species-specific traits. This gilthead sea bream feature was concurrent with a clear habituation to high densities, which was evidenced by a shorter recovery time of physical activity and respiration following repeated stress confinement test. This adaptive feature occurred simultaneously with a shift in white skeletal muscle from systemic to local growth regulation that also primed muscle regeneration through main changes in the expression pattern of growth hormone receptors and myogenic factors. These adjustments were considered adaptive in an environment with an enhanced risk of involuntary fish collisions that favour a higher abundance of slight external injuries. Ultimately, such integrative approach will contribute to better defining the maximum stocking density and will also serve to provide practical guidelines to update fish welfare auditing in order to ensure that fish welfare and production goals are aligned.

## Figures and Tables

**Table 1:** PCR array layout for white skeletal muscle gene expression profiling, including markers of GH/IGF system, muscle cell growth, immune response, oxidative metabolism and energy sensing, and antioxidant defence. Specific PCR primer pair sequences are listed in Supplementary Table S1.

Function	Gene	Symbol	GenBank
GH/IGF SYSTEM	Growth hormone receptor type 1	*ghr1*	AF438176
	Growth hormone receptor type 2	*ghr2*	AY573601
	Insulin-like growth factor 1	*igf1*	AY996779
	Insulin-like growth factor 2	*igf2*	AY996778
	Insulin-like growth factor binding protein 3a	*igfbp3a*	MH577191
	Insulin-like growth factor binding protein 3b	*igfbp3b*	MH577192
	Insulin-like growth factor binding protein 5a	*igfbp5a*	MH577193
	Insulin-like growth factor binding protein 5b	*igfbp5b*	MH577194
	Insulin-like growth factor binding protein 6a	*igfbp6a*	MH577195
	Insulin-like growth factor binding protein 6b	*igfbp6b*	MH577196
MUSCLE CELL GROWTH	Myoblast determination protein 1	*myod1*	AF478568
	Myogenic determination protein 2	*myod2*	AF478569
	Myogenic factor 5	*myf5*	JN034420
	Myogenic factor 6	*myf6/mrf4*	JN034421
	Myostatin	*mstn*	AF258448
	Myocyte-specific enhancer factor 2a	*mef2a*	KM522777
	Myocyte-specific enhancer factor 2c	*mef2c*	KM522778
	Follistatin	*fst*	AY544167
IMMUNE RESPONSE	Interleukin 1*β*	*il1β*	AJ419178
	Interleukin 6	*il6*	EU244588
	Interleukin 8	*il8*	JX976619
	Interleukin 10	*il10*	JX976621
	Interleukin 12 subunit *β*	*il12β*	JX976624
OXIDATIVE METABOLISM & ENERGY SENSING	Hypoxia inducible factor 1*α*	*hif1α*	JQ308830
	Proliferator-activated receptor γ coactivator 1*α*	*pgc1α*	JX975264
	Proliferator-activated receptor γ coactivator 1*β*	*pgc1β*	JX975265
	Carnitine palmitoyltransferase 1a	*cpt1a*	JQ308822
	Citrate synthase	*cs*	JX975229
	NADH-ubiquinone oxidoreductase chain 2	*nd2*	KC217558
	NADH-ubiquinone oxidoreductase chain 5	*nd5*	KC217559
	Cytochrome c oxidase subunit 1	*cox1*	KC217652
	Cytochrome c oxidase subunit 2	*cox2*	KC217653
	Uncoupling protein 3	*ucp3*	EU555336
	Sirtuin1	*sirt1*	KF018666
	Sirtuin2	*sirt2*	KF018667
ANTIOXIDANT DEFENCE	Catalase	*cat*	JQ308823
	Glutathione peroxidase 4	*gpx4*	AM977818
	Glutathione reductase	*gr*	AJ937873
	Peroxiredoxin 3	*prdx3*	GQ252681
	Peroxiredoxin 5	*prdx5*	GQ252683
	Superoxide dismutase [Mn]	*mn-sod/sod2*	JQ308833
	Glucose-regulated protein 170 kDa	*grp170*	JQ308821
	Glucose-regulated protein 94 kDa	*grp94*	JQ308820
	Glucose-regulated protein 75 kDa	*grp75*	DQ524993

**Table 2:** Data on growth performance, muscle fat deposition and haematological parameters in gilthead sea bream reared at two different stocking densities from June (CTRL 10 kg/m^3^; HD, 18 kg/m^3^) to beginning of August 2022 (CTRL 15 kg/m^3^; HD, 24 kg/m^3^). Data on growth performance are the mean ± SEM of duplicate tanks. Data on fat deposition and haematological parameters are the mean ± SEM of 15–19 fish. Statistically significant differences are analysed by Student’s *t*-test.

	CTRL (10–15 kg/m^3^)	HD (18–24 kg/m^3^)	*p * ^1^
Initial body weight (g)	441.96 ± 6.80	427.92 ± 4.57	0.079
Initial body length (cm)	25.20 ± 0.12	25.06 ± 0.09	0.278
Feed intake (g dry matter/fish)	310.26 ± 2.18	252.19 ± 2.26	<0.001
Final body weight (g)	639.17 ± 8.08	574.74 ± 5.68	<0.001
Final body length (cm)	28.62 ± 0.13	27.97 ± 0.09	<0.001
Final CFK ^2^	2.72 ± 0.02	2.62 ± 0.01	<0.001
Liver weight (g)	7.05 ± 0.40	5.01 ± 0.52	0.007
HSI (%) ^3^	1.14 ± 0.05	0.91 ± 0.09	0.036
SGR (%) ^4^	0.60 ± 0.01	0.47 ± 0.01	<0.001
FCR ^5^	1.57 ± 0.02	1.72 ± 0.01	0.022
Haemoglobin (g/dL)	10.17 ± 0.30	8.89 ± 0.26	0.006
Haematocrit (%)	49.16 ± 1.74	38.09 ± 2.25	<0.001
Muscle fat (%) ^6^	12.03 ± 0.42	10.47 ± 0.38	0.011

^1^ Student’s *t*-test, *p*-value. ^2^ Fulton’s body condition factor: CFK = 100 × (body weight/standard length^3^). ^3^ Hepatosomatic index: HIS = 100 × (liver weight/fish weight). ^4^ Specific growth rate: SGR = 100 × (ln final body weight − ln initial body weight)/days. ^5^ Feed conversion ratio: FCR = 100 × (dry feed intake/wet weight gain). ^6^ Fat-meter measurements.

## Data Availability

Data are contained within the article and Supplementary Materials.

## Supplementary Materials

The following supporting information can be downloaded at: https://www.mdpi.com/article/10.3390/biology13110879/s1, Supplementary Table S1. Primers for qPCR amplification of white skeletal muscle genes; Supplementary Table S2. Relative gene expression of white skeletal muscle mRNA transcripts in CTRL (15 kg/m^3^) and HD (24 kg/m^3^) fish. Values are the mean ± SEM of 8 fish. All data are in reference to the expression level of *fst* in CTRL fish with an arbitrary assigned value of 1. Student’s *t*-test was used to determine significant differences between experimental conditions (*p* < 0.05). Differentially expressed genes are in bold.

## References

[1] Garlock T., Asche F., Anderson J., Bjørndal T., Kumar G., Lorenzen K., Ropicki A., Smith M., Tveterås R.A. (2019). Global Blue Revolution: Aquaculture Growth Across Regions, Species, and Countries. Rev. Fish. Sci. Aquac..

[2] Masson-Delmotte V., Zhai P., Pirani S., Connors C., Péan S., Berger N., Caud Y., Chen L., Goldfarb M.I., Scheel Monteiro P.M., Masson-Delmotte V., Zhai P., Pirani A., Connors S.L., Péan C., Berger S., Caud N., Chen Y., Goldfarb L., Gomis M.I. (2021). IPCC, 2021: Summary for Policymakers. Climate Change 2021: The Physical Science Basis. Contribution of Working Group I to the Sixth Assessment Report of the Intergovernmental Panel on Climate Change.

[3] Ellis T., North B., Scott A.P., Bromage N.R., Porter M., Gadd D. (2002). The relationships between stocking density and welfare in farmed rainbow trout. J. Fish Biol..

[4] North B.P., Turnbull J.F., Ellis T., Porter M.J., Migaud H., Bron J., Bromage N.R. (2006). The impact of stocking density on the welfare of rainbow trout (*Oncorhynchus mykiss*). Aquaculture.

[5] Baldwin L. (2011). The effects of stocking density on fish welfare. Plymouth Stud. Sci..

[6] Jia R., Liu B., Feng W.R., Han C., Huang B., Lei J.L. (2016). Stress and immune responses in skin of turbot (*Scophthalmus maximus*) under different stocking densities. Fish Shellfish Immunol..

[7] Liu B., Liu Y., Sun G. (2017). Effects of stocking density on growth performance and welfare-related physiological parameters of Atlantic salmon *Salmo salar* L. recirculating aquaculture system. Aquac. Res..

[8] Wu F., Wen H., Tian J., Jiang M., Liu W., Yang C., Yu L., Lu X. (2018). Effect of stocking density on growth performance, serum biochemical parameters, and muscle texture properties of genetically improved farm tilapia, *Oreochromis niloticus*. Aquac. Int..

[9] Browman H.I., Cooke S.J., Cowx I.G., Derbyshire S.W., Kasumyan A., Key B., Rose J., Schwab A., Skiftesvik A.B., Stevens D. (2019). Welfare of aquatic animals: Where things are, where they are going, and what it means for research, aquaculture, recreational angling, and commercial fishing. ICES J. Mar. Sci..

[10] Herman J.P. (2013). Neural control of chronic stress adaptation. Front. Behav. Neurosci..

[11] Galhardo L., Oliveira R.F. (2009). Psychological stress and welfare in fish. Ann. Rev. Biomed. Sci..

[12] Schreck C.B., Tort L. (2016). The concept of stress in fish. Fish Physiol..

[13] Winberg S., Sneddon L. (2022). Impact of intraspecific variation in teleost fishes: Aggression, dominance status and stress physiology. J. Exp. Biol..

[14] Selye H. (1976). The stress concept. Can. Med. Assoc. J..

[15] Koolhaas J.M., Bartolomucci A., Buwalda B., de Boer S.F., Flügge G., Korte S.M., Meerlo P., Murison R., Olivier B., Palanza P. (2011). Stress revisited: A critical evaluation of the stress concept. Neurosci. Biobehav. Rev..

[16] Perera E., Rosell-Moll E., Martos-Sitcha J.A., Naya-Català F., Simó-Mirabet P., Calduch-Giner J.A., Manchado M., Afonso J.M., Pérez-Sánchez J. (2021). Physiological trade-offs associated with fasting weight loss, resistance to exercise and behavioral traits in farmed gilthead sea bream (*Sparus aurata*) selected by growth. Aquac. Rep..

[17] Naya-Català F., Martos-Sitcha J.A., de las Heras V., Simó-Mirabet P., Calduch-Giner J.À., Pérez-Sánchez J. (2021). Targeting the mild-hypoxia driving force for metabolic and muscle transcriptional reprogramming of gilthead sea bream (*Sparus aurata*) juveniles. Biology.

[18] Iwama G.K., Afonso L.O., Vijayan M.M. (1998). Stress in fish. Ann. N. Y. Acad. Sci..

[19] Barton B.A. (2002). Stress in fishes: A diversity of responses with particular reference to changes in circulating corticosteroids. Integr. Comp. Biol..

[20] Folkedal O., Fernö A., Nederlof M.A., Fosseidengen J.E., Cerqueira M., Olsen R.E., Nilsson J. (2018). Habituation and conditioning in gilthead sea bream (*Sparus aurata*): Effects of aversive stimuli, reward and social hierarchies. Aquac. Res..

[21] Sopinka N.M., Donaldson M.R., O’Connor C.M., Suski C.D., Cooke S.J. (2016). Stress indicators in fish. Fish Physiol..

[22] Sadoul B., Geffroy B. (2019). Measuring cortisol, the major stress hormone in fishes. J. Fish Biol..

[23] Noble C., Gismervik K., Iversen M.H., Kolarevic J., Nilsson J., Stien L.H., Turnbull J.F. (2020). Welfare Indicators for Farmed Rainbow Trout: Tools for Assessing Fish Welfare.

[24] Fazio F. (2019). Fish hematology analysis as an important tool of aquaculture: A review. Aquaculture.

[25] Holhorea P.G., Naya-Català F., Belenguer Á., Calduch-Giner J.A., Pérez-Sánchez J. (2023). Understanding how high stocking densities and concurrent limited oxygen availability drive social cohesion and adaptive features in regulatory growth, antioxidant defense and lipid metabolism in farmed gilthead sea bream (*Sparus aurata*). Front. Physiol..

[26] Naya-Català F., Torrecillas S., Piazzon M.C., Sarih S., Calduch-Giner J., Fontanillas R., Hostins B., Sitjà-Bobadilla A., Acosta F., Pérez-Sánchez J. (2024). Can the genetic background modulate the effects of feed additives? Answers from gut microbiome and transcriptome interactions in farmed gilthead sea bream (*Sparus aurata*) fed with a mix of phytogenics, organic acids or probiotics. Aquaculture.

[27] Barreto M.O., Rey Planellas S., Yang Y., Phillips C., Descovich K. (2022). Emerging indicators of fish welfare in aquaculture. Rev. Aquac..

[28] Calduch-Giner J., Holhorea P.G., Ferrer M.Á., Naya-Català F., Rosell-Moll E., Vega García C., Prunet P., Espmark A.M., Leguen I., Kolarevic J. (2022). Revising the impact and prospects of activity and ventilation rate bio-loggers for tracking welfare and fish-environment interactions in salmonids and Mediterranean farmed fish. Front. Mar. Sci..

[29] Li X., Hao Y., Akhter M., Li D. (2022). A novel automatic detection method for abnormal behavior of single fish using image fusion. Comput. Electron. Agric..

[30] Li D., Du Z., Wang Q., Wang J., Du L. (2024). Recent advances in acoustic technology for aquaculture: A review. Rev. Aquac..

[31] Castanheira M.F., Cerqueira M., Millot S., Gonçalves R.A., Oliveira C.C.V., Conceiçao L.E.C., Martins C.I.M. (2016). Are personality traits consistent in fish? The influence of social context. Appl. Anim. Behav. Sci..

[32] Carbonara P., Alfonso S., Zupa W., Manfrin A., Fiocchi E., Pretto T., Spedicato M.T., Lembo G. (2019). Behavioural and physiological responses to stocking density in sea bream (*Sparus aurata*): Do coping styles matter?. Physiol. Behav..

[33] Castanheira M.F., Conceição L.E., Millot S., Rey S., Bégout M.L., Damsgard B., Kristiansen T., Höglund E., Overli O., Martins C.I. (2017). Coping styles in farmed fish: Consequences for aquaculture. Rev. Aquac..

[34] Arechavala-Lopez P., Nazzaro-Alvarez J., Jardí-Pons A., Reig L., Carella F., Carrassón M., Roque A. (2020). Linking stocking densities and feeding strategies with social and individual stress responses on gilthead sea bream (*Sparus aurata*). Physiol. Behav..

[35] Toxqui-Rodríguez S., Holhorea P.G., Naya-Català F., Calduch-Giner J.À., Sitjà-Bobadilla A., Piazzon C., Pérez-Sánchez J. (2024). Differential reshaping of skin and intestinal microbiota by stocking density and oxygen availability in farmed gilthead sea bream (*Sparus aurata*): A behavioral and network-based integrative approach. Microorganisms.

[36] Fanouraki E., Mylonas C.C., Papandroulakis N., Pavlidis M. (2011). Species specificity in the magnitude and duration of the acute stress response in Mediterranean marine fish in culture. General Comp. Endocrinol..

[37] Conde-Sieira M., Valente L.M., Hernandez-Perez J., Soengas J.L., Míguez J.M., Gesto M. (2018). Short-term exposure to repeated chasing stress does not induce habituation in Senegalese sole, *Solea senegalensis*. Aquaculture.

[38] Madaro A., Olsen R.E., Kristiansen T.S., Ebbesson L.O., Flik G., Gorissen M. (2016). A comparative study of the response to repeated chasing stress in Atlantic salmon (*Salmo salar* L.) parr and post-smolts. Comp. Biochem. Physiol. Part A Mol. Integr. Physiol..

[39] Haukenes A.H., Barton B.A. (2004). Characterization of the cortisol response following an acute challenge with lipopolysaccharide in yellow perch and the influence of rearing density. J. Fish Biol..

[40] Di Marco P., Priori A., Finoia M.G., Massari A., Mandich A., Marino G. (2008). Physiological responses of European sea bass *Dicentrarchus labrax* to different stocking densities and acute stress challenge. Aquaculture.

[41] Bosch-Belmar M., Giomi F., Rinaldi A., Mandich A., Fuentes V., Mirto S., Sarà G., Piraino S. (2016). Concurrent environmental stressors and jellyfish stings impair caged European sea bass (*Dicentrarchus labrax*) physiological performances. Sci. Rep..

[42] Hoyle I., Oidtmann B., Ellis T., Turnbull J., North B., Nikolaidis J., Knowles T.G. (2007). A validated macroscopic key to assess fin damage in farmed rainbow trout (*Oncorhynchus mykiss*). Aquaculture.

[43] Martos-Sitcha J.A., Sosa J., Ramos-Valido D., Bravo F.J., Carmona-Duarte C., Gomes H.L., Calduch-Giner J.A., Cabruja E., Vega A., Ferrer M.A. (2019). Ultra-low power sensor devices for monitoring physical activity and respiratory frequency in farmed fish. Front. Physiol..

[44] Ferrer M.A., Calduch-Giner J.A., Díaz M., Sosa J., Rosell-Moll E., Santana Abril J., Santana Sosa G., Delgado T.B., Carmona C., Martos-Sitcha J.A. (2020). From operculum and body tail movements to different coupling of physical activity and respiratory frequency in farmed gilthead sea bream and European sea bass. Insights on aquaculture biosensing. Comput. Electron. Agric..

[45] Livak K.J., Schmittgen T.D. (2001). Analysis of relative gene expression data using real-time quantitative PCR and the 2(-delta delta CT) method. Methods.

[46] Romagnoli C., Iantomasi T., Brandi M.L. (2021). Available in vitro models for human satellite cells from skeletal muscle. Int. J. Mol. Sci..

[47] Braithwaite V.A., Ebbesson L.O.E. (2014). Pain and stress responses in farmed fish. Rev. Sci. Tech..

[48] Martos-Sitcha J.A., Mancera J.M., Prunet P., Magnoni L.J. (2020). Welfare and stressors in fish: Challenges facing aquaculture. Front. Physiol..

[49] Menon S.V., Kumar A., Middha S.K., Paital B., Mathur S., Johnson R., Kademan A., Usha T., Hemavathi K.N., Dayal S. (2023). Water physicochemical factors and oxidative stress physiology in fish, a review. Front. Environ. Sci..

[50] Raposo de Magalhães C.S.F., Cerqueira M.A.C., Schrama D., Moreira M.J.V., Boonanuntanasarn S., Rodrigues P.M.L. (2020). A Proteomics and other Omics approach in the context of farmed fish welfare and biomarker discovery. Rev. Aquac..

[51] Araújo-Luna R., Ribeiro L., Bergheim A., Pousão-Ferreira P. (2018). The impact of different rearing condition on gilthead seabream welfare: Dissolved oxygen levels and stocking densities. Aquac. Res..

[52] Berger J. (2010). Fear-mediated food webs. Trophic Cascades: Predators, Prey, and the Changing Dynamics of Nature.

[53] de Matos Dias D., de Campos C.B., Guimarães Rodrigues F.H. (2018). Behavioural ecology in a predator-prey system. Mamm. Biol..

[54] Carbonara P., Dioguardi M., Cammarata M., Zupa W., Vazzana M., Spedicato M.T., Lembo G. (2019). Basic knowledge of social hierarchies and physiological profile of reared sea bass *Dicentrarchus labrax* (L.). PLoS ONE.

[55] Calduch-Giner J., Rosell-Moll E., Besson M., Vergnet A., Bruant J.S., Clota F., Holhorea P.G., Allal F., Vandeputte M., Pérez-Sánchez J. (2023). Changes in transcriptomic and behavioural traits in activity and ventilation rates associated with divergent individual feed efficiency in gilthead sea bream (*Sparus aurata*). Aquac. Rep..

[56] Samaras A., Espírito Santo C., Papandroulakis N., Mitrizakis N., Pavlidis M., Höglund E., Pelgrim T.N.M., Zethof J., Spanings F.A.T., Vindas M.A. (2018). Allostatic load and stress physiology in European seabass (*Dicentrarchus labrax* L.) and gilthead seabream (*Sparus aurata* L.). Front. Endocrin..

[57] Montero D., Izquierdo M.S., Tort L., Robaina L., Vergara J.M. (1999). High stocking density produces crowding stress altering some physiological and biochemical parameters in gilthead seabream, *Sparus aurata*, juveniles. Fish Physiol. Biochem..

[58] Sangiao-Alvarellos S., Guzmán J.M., Láiz-Carrión R., Míguez J.M., Martín Del Río M.P., Mancera J.M., Soengas J.L. (2005). Interactive effects of high stocking density and food deprivation on carbohydrate metabolism in several tissues of gilthead sea bream *Sparus auratus*. J. Exp. Zool. A Comp. Exp. Biol..

[59] Martos-Sitcha J.A., Simó-Mirabet P., de Las Heras V., Calduch-Giner J.À., Pérez-Sánchez J. (2019). Tissue-specific orchestration of gilthead sea bream resilience to hypoxia and high stocking density. Front. Physiol..

[60] López-Patiño M.A., Skrzynska A.K., Naderi F., Mancera J.M., Míguez J.M., Martos-Sitcha J.A. (2021). High stocking density and food deprivation increase brain monoaminergic activity in gilthead sea bream (*Sparus aurata*). Animals.

[61] Li L., Shen Y., Yang W., Xu X., Li J. (2021). Effect of different stocking densities on fish growth performance: A meta-analysis. Aquaculture.

[62] Farhat E., Cheng H., Romestaing C., Pamenter M., Weber J.M. (2021). Goldfish response to chronic hypoxia: Mitochondrial respiration, fuel preference and energy metabolism. Metabolites.

[63] Wang Z., Pu D., Zheng J., Li P., Lü H., Wei X., Li M., Li D., Gao L. (2023). Hypoxia-induced physiological responses in fish: From organism to tissue to molecular levels. Ecotoxicol. Environ. Saf..

[64] Gorr T.A. (2017). Hypometabolism as the ultimate defence in stress response: How the comparative approach helps understanding of medically relevant questions. Acta Physiol..

[65] Ruan W., Ji W.W., Zheng L., Yue D.D., Fang H. (2020). On hypoxia stress in fish and its nutritional regulation and response. Mar. Fish..

[66] Saravanan S., Geurden I., Figueiredo-Silva A.C., Kaushik S.J., Haidar M.N., Verreth J.A., Schrama J.W. (2012). Control of voluntary feed intake in fish: A role for dietary oxygen demand in Nile tilapia (*Oreochromis niloticus*) fed diets with different macronutrient profiles. Br. J. Nutr..

[67] Goldan O., Popper D., Karplus I. (2003). Food competition in small groups of juvenile gilthead seabream (*Sparus aurata*). Isr. J. Aquac. Bamidgeh.

[68] Montero D., Lalumera G., Izquierdo M.S., Caballero M.J., Saroglia M., Tort L. (2009). Establishment of dominance relationships in gilthead sea bream *Sparus aurata* juveniles during feeding: Effects on feeding behaviour, feed utilization and fish health. J. Fish. Biol..

[69] Arechavala-Lopez P., Diaz-Gil C., Saraiva J.L., Moranta D., Castanheira M.F., Nuñez-Velázquez S., Ledesma-Corvi S., Mora-Ruiz M.R., Grau A. (2019). Effects of structural environmental enrichment on welfare of juvenile seabream (*Sparus aurata*). Aquac. Rep..

[70] Oikonomidou E., Batzina A., Karakatsouli N. (2019). Effects of food quantity and distribution on aggressive behaviour of gilthead seabream and European seabass. Appl. Anim. Behav. Sci..

[71] Parma L., Pelusio N.F., Gisbert E., Esteban M.A., D’Amico F., Soverini M., Candela M., Dondi F., Gatta P.P., Bonaldo A. (2020). Effects of rearing density on growth, digestive conditions, welfare indicators and gut bacterial community of gilthead sea bream (*Sparus aurata* L. 1758) fed different fishmeal and fish oil dietary levels. Aquaculture.

[72] Otero-Tarrazón A., Perelló-Amorós M., Jorge-Pedraza V., Moshayedi F., Sánchez-Moya A., García-Pérez I., Fernández-Borràs J., García de la Serrana D., Navarro I., Blasco J. (2023). Muscle regeneration in gilthead sea bream: Implications of endocrine and local regulatory factors and the crosstalk with bone. Front. Endocrinol..

[73] Pérez-Sánchez J., Simó-Mirabet P., Naya-Català F., Martos-Sitcha J.A., Perera E., Bermejo-Nogales A., Benedito-Palos L., Calduch-Giner J.A. (2018). Somatotropic axis regulation unravels the differential effects of nutritional and environmental factors in growth performance of marine farmed fishes. Front. Endocrinol..

[74] Zammit P.S. (2017). Function of the myogenic regulatory factors Myf5, MyoD, Myogenin and MRF4 in skeletal muscle, satellite cells and regenerative myogenesis. Semin. Cell Dev. Biol..

[75] Hernández-Hernández J.M., García-González E.G., Brun C.E., Rudnicki M.A. (2017). The myogenic regulatory factors, determinants of muscle development, cell identity and regeneration. Semin. Cell Dev. Biol..

[76] White L.J., Russell A.J., Pizzey A.R., Dasmahapatra K.K., Pownall M.E. (2023). The presence of two MyoD genes in a subset of Acanthopterygii fish is associated with a polyserine insert in MyoD1. J. Dev. Biol..

[77] McFarland D.C., Velleman S.G., Pesall J.E., Liu C. (2006). Effect of myostatin on turkey myogenic satellite cells and embryonic myoblasts. Comp. Biochem. Physio. A Mol. Integr. Physiol..

[78] Sharma M., Langley B., Bass J., Kambadur R. (2001). Myostatin in muscle growth and repair. Exerc. Sport Sci. Rev..

[79] Langley B., Thomas M., Bishop A., Sharma M., Gilmour S., Kambadur R. (2002). Myostatin inhibits myoblast differentiation by down-regulating MyoD expression. J. Biol. Chem..

[80] Gesto M. (2019). Consistent individual competitive ability in rainbow trout as a proxy for coping style and its lack of correlation with cortisol responsiveness upon acute stress. Physiol. Behav..

[81] Champneys T., Castaldo G., Consuegra S., Garcia de Leaniz C. (2018). Density-dependent changes in neophobia and stress-coping styles in the world’s oldest farmed fish. R. Soc. Open Sci..

[82] Barton B.A., Ribas L., Acerete L., Tort L. (2005). Effects of chronic confinement on physiological responses of juvenile gilthead sea bream, *Sparus aurata* L., to acute handling. Aquac. Res..

[83] Brown C. (2015). Fish intelligence, sentience and ethics. Anim. Cogn..

[84] Kohda M., Hotta T., Takeyama T., Awata S., Tanaka H., Asai J.Y., Jordan A.L. (2019). If a fish can pass the mark test, what are the implications for consciousness and self-awareness testing in animals?. PLoS Biol..

[85] Nilsson J., Stien L.H., Fosseidengen J.E., Olsen R.E., Kristiansen T.S. (2012). From fright to anticipation: Reward conditioning versus habituation to a moving dip net in farmed Atlantic cod (*Gadus morhua*). Appl. Anim. Behav. Sci..

[86] Koakoski G., Kreutz L.C., Fagundes M., Oliveira T.A., Ferreira D., Rosa J.G.S.D., Barcellos L.J.G. (2013). Repeated stressors do not provoke habituation or accumulation of the stress response in the catfish *Rhamdia quelen*. Neotrop. Ichthyol..

[87] Bratland S., Stien L.H., Braithwaite V.A., Juell J.E., Folkedal O., Nilsson J., Oppedal F., Fosseidengen J.E., Kristiansen T.S. (2010). From fright to anticipation: Using aversive light stimuli to investigate reward conditioning in large groups of Atlantic salmon (*Salmo salar*). Aquac. Int..

[88] Ruiz N., García-Meilán I., Khansari A.R., Teles M., Pastor J., Tort L. (2024). Repeated hypoxic episodes allow hematological and physiological habituation in rainbow trout. Front. Physiol..

[89] Fernandes-de-Castilho M., Pottinger T.G., Volpato G.L. (2008). Chronic social stress in rainbow trout: Does it promote physiological habituation?. Gen. Comp. Endocrinol..

[90] Jesus J., Amorim M.C.P., Fonseca P.J., Teixeira A., Natário S., Carrola J., Varandas S., Perreira L.T., Cortes R.M. (2019). Acoustic barriers as an acoustic deterrent for native potamodromous migratory fish species. J. Fish Biol..

[91] Macaulay G., Bui S., Oppedal F., Dempster T. (2021). Challenges and benefits of applying fish behaviour to improve production and welfare in industrial aquaculture. Rev. Aquac..

