# Supplementary material for: Behavioural Monitoring Underlines Habituation to Repeated Stressor Stimuli in Farmed Gilthead Sea Bream (Sparus aurata) Reared at a High Stocking Density
Source: Biology (Basel). 2024 Oct 29;13(11):879. doi: 10.3390/biology13110879 (PMC11592208; doi:10.3390/biology13110879)
Supplement: Supplementary file 1 [file biology-13-00879-s001.zip › biology-3274850-supplementary.pdf]

Table S1. Primers for qPCR amplification of white skeletal muscle genes.

Gene	Symbol	GenBank	Primer
Growth hormone receptor-type 1	*ghr1*	AF438176	F: ACC TGT CAG CCA CCA CAT GA<br>R: TCG TGC AGA TCT GGG TCG TA
Growth hormone receptor-type 2	*ghr2*	AY573601	F: GAG TGA ACC CGG CCT GAC AG<br>R: GCG GTG GTA TCT GAT TCA TGG T
Insulin-like growth factor 1	*igf1*	AY996779	F: TGT CTA GCG CTC TTT CCT TTC A<br>R: AGA GGG TGT GGC TAC AGG AGA TAC
Insulin-like growth factor 2	*igf2*	AY996778	F: TGG GAT CGT AGA GGA GTG TTG T<br>R: CTG TAG AGA GGT GGC CGA CA
Insulin-like growth factor binding protein 3a	*igfbp3a*	MH577191	F: ACA GGC GTG TGG AGT GTA<br>R: TGG TGC TGG CAG GTC AAG
Insulin-like growth factor binding protein 3b	*igfbp3b*	MH577192	F: GCC AGA TTA TGG TCC CTG TCG GAG AGA G<br>R: GTC TGT AAT CTT GAG GCT GCT GAG GAT GCT
Insulin-like growth factor binding protein 5a	*igfbp5a*	MH577193	F: GAA TCT CAC GAT GAC GCC AT<br>R: TGC TGA TGT GGT CTC TTC C
Insulin-like growth factor binding protein 5b	*igfbp5b*	MH577194	F: GCA AGC AGT GTA AGC CAT CTC<br>R: TGA ACG CCG TAC TTG TCC A
Insulin-like growth factor binding protein 6a	*igfbp6a*	MH577195	F: CGA CCC GAA TCA CGA CAT ATA CAT<br>R: ACT TGC CAC GCC GCT TAC
Insulin-like growth factor binding protein 6b	*igfbp6b*	MH577196	F: GAT TGC TCA CTG CGG ATC<br>R: GGA GGG ACA GAC CTT GAA
Myoblast determination protein 1	*myod1*	AF478568	F: ATG GAG CTG TCG GAT ATC TCT TTC<br>R: GAA GCA GGG GTC ATC GTA GAA ATC
Myogenic determination protein 2	*myod2*	AF478569	F: CCA ACT GCT CTG ATG GCA TGA TGG ATT TC<br>R: GAC CGT TTG CTT CTC CTG GAC TCG TAT G
Myogenic factor 5	*myf5*	JN034420	F: GCA TGG TTG ACA GCA ACA GTC CAG TGT<br>R: TGT CTT ATC GCC CAA AGT GTC GTT CTT CAT
Myogenic factor 6	*myf6/mrf4*	JN034421	F: GCA GCA ATG ACA AAC CAG AGA GAC GGA ACA<br>R: GAG GCT GGA GGA CGC CGA AGA TTC A
Myostatin	*mstn*	AF258448	F: AAG AGC AGA TCA TCT ACG GCA AGA TCC<br>R: TCA AGA GCA TCC ACA ACG GTC TAC CA
Myocyte-specific enhancer factor 2a	*mef2a*	KM522777	F: ATG GAC GAG AGG AAC AGG CAG GTT A<br>R: GGC TAT CTC ACA GTC ACA TAG TAC GCT CAG
Myocyte-specific enhancer factor 2c	*mef2c*	KM522778	F: TAG CAA CTC CCA CTC TAC CAG GAC AAG<br>R: GGA ATA CTC GGC ACC ATA AGA AGT CG
Follistatin	*fst*	AY544167	F: GGA CCA GAC AAA CAA CGC ATA TTG<br>R: CAT AGA TGA TCC CGT CGT TTC CAC
Interleukin 1β	*il1β*	AJ419178	F: GCG ACC TAC CTG CCA CCT ACA CC<br>R: TCG TCC ACC GCC TCC AGA TGC

Interleukin 6	*il6*	EU244588	F: TCT TGA AGG TGG TGC TGG AAG TG<br>R: AAG GAC AAT CTG CTG GAA GTG AGG
Interleukin 8	*il8*	JX976619	F: CAG CAG AGT CTT CAT CGT CAC TAT TG<br>R: AGG CTC GCT TCA CTG ATG G
Interleukin 10	*il10*	JX976621	F: AAC ATC CTG GGC TTC TAT CTG<br>R: GTG TCC TCC GTC TCA TCT G
Interleukin 12 subunit *β*	*il12β*	JX976624	F: ATT CCC TGT GTG GTG GCT GCT<br>R: GCT GGC ATC CTG GCA CTG AAT
Hypoxia inducible factor 1*α*	*hif1α*	JQ308830	F: CAG ATG AGC CTC TAA CTT GTG GAC<br>R: TTA GCA AGA ATG GTG GCA AGA TGA G
Proliferator-activated receptor γ coactivator 1*α*	*pgc1α*	JX975264	F: CGT GGG ACA GGT GTA ACC AGG ACT C<br>R: ACC AAC CAA GGC AGC ACA CTC TAA TTC T
Proliferator-activated receptor γ coactivator 1*β*	*pgc1β*	JX975265	F: TCA GAG GAA GAG GCG GAT<br>R: GAC ACA GGT GGA GGA TGG
Carnitine palmitoyltransferase 1a	*cpt1a*	JQ308822	F: GTG CCT TCG TTC GTT CCA TGA TC<br>R: TGA TGC TTA TCT GCT GCC TGT TTG
Citrate synthase	*cs*	JX975229	F: TCC AGG AGG TGA CGA GCC<br>R: GTG ACC AGC AGC CAG AAG AG
NADH-ubiquinone oxidoreductase chain 2	*nd2*	KC217558	F: TAG GTT GAA TGA CCA TCG TA<br>R: GGC TAA GGA GTT GAG GTT
NADH-ubiquinone oxidoreductase chain 5	*nd5*	KC217559	F: CCT AAA CGC CTG AGC CCT GG<br>R: GCT GTA AAC GAG GTG GCT AGA AGG
Cytochrome c oxidase subunit 1	*cox1*	KC217652	F: GTC CTA CTT CTT CTG TCC CTT CCT GTT CT<br>R: AGG TTT CGG TCT GTA AGG AGC ATT GTA ATC
Cytochrome c oxidase subunit 2	*cox2*	KC217653	F: ACT GCC TAC ACA GGA CCT TGC C<br>R: GTC TGC TTC CAG GAG ACG GAA TTG T
Uncoupling protein 3	*ucp3*	EU555336	F: AGG TGC GAC TGG CTG ACG<br>R: TTC GGC ATA CAA CCT CTC CAA AG
Sirtuin1	*sirt1*	KF018666	F: GGT TCC TAC AGT TTC ATC CAG CAG CAC ATC<br>R: CCT CAG AAT GGT CCT CGG ATC GGT CTC
Sirtuin2	*sirt2*	KF018667	F: GAA CAA TCC GAC GAC AGC AGT GAA G<br>R: AGG TTA CGC AGG AAG TCC ATC TCT
Catalase	*cat*	JQ308823	F: TGG TCG AGA ACT TGA AGG CTG TC<br>R: AGG ACG CAG AAA TGG CAG AGG
Glutathione peroxidase 4	*gpx4*	AM977818	F: TGC GTC TGA TAG GGT CCA CTG TC<br>R: GTC TGC CAG TCC TCT GTC GG
Glutathione reductase	*gr*	AJ937873	F: TGT TCA GCC ACC CAC CCA TCG G<br>R: GCG TGA TAC ATC GGA GTG AAT GAA GTC TTG
Peroxiredoxin 3	*prdx3*	GQ252681	F: ATC AAC ACC CCA CGC AAG ACT G<br>R: ACC GTT TGG ATC AAT GAG GAA CAG ACC
Peroxiredoxin 5	*prdx5*	GQ252683	F: GAG CAC GGA ACA GAT GGC AAG G<br>R: TCC ACA TTG ATC TTC TTC ACG ACT CC
Superoxide dismutase [Mn]	*mn-sod / sod2*	JQ308833	F: CCT GAC CTG ACC TAC GAC TAT GG

			R: AGT GCC TCC TGA TAT TTC TCC TCT G
Glucose-regulated protein 170 kDa	*grp170*	JQ308821	F: CAG AGG AGG CAG ACA GCA AGA C<br>R: TTC TCA GAC TCA GCA TTT CCA GAT TTC
Glucose-regulated protein 94 kDa	*grp94*	JQ308820	F: AAG GCA CAG GCT TAC CAG ACA G<br>R: CTT CAG CAT CAT CGC CGA CTT TC
Glucose-regulated protein 75 kDa	*grp75*	DQ524993	F: TCC GGT GTG GAT CTG ACC AAA GAC<br>R: TGT TTA GGC CCA GAA GCA TCC ATG
Beta-actin	*actb*	KY388508	F: TCC TGC GGA ATC CAT GAG A<br>R: GAC GTC GCA CTT CAT GAT GCT

Table S2. Relative gene expression of white skeletal muscle mRNA transcripts in CTRL (15 kg/m^3) and HD (24 kg/m^3) fish. Values are the mean ± SEM of 8 fish. All data are in reference to the expression level of *fst* in CTRL fish with an arbitrary assigned value of 1. Student's t-test was used to determine significant differences between experimental conditions ($p < 0.05$). Differentially expressed genes are in bold.

	**CTRL (15)**	**HD (25)**	***p*** [1]
ghr1	3.21 ± 0.27	2.55 ± 0.23	0.092
ghr2	**0.97 ± 0.11**	**1.37 ± 0.14**	**0.044**
igf1	0.13 ± 0.02	0.12 ± 0.01	0.681
igf2	0.96 ± 0.07	1.05 ± 0.07	0.363
igfbp3a	3.59 ± 0.49	3.68 ± 0.34	0.886
igfbp3b	0.005 ± 0.00	0.004 ± 0.00	0.486
igfbp5a	0.58 ± 0.12	0.56 ± 0.06	0.919
igfbp5b	4.06 ± 0.43	3.77 ± 0.34	0.614
igfbp6a	0.07 ± 0.01	0.08 ± 0.01	0.842
igfbp6b	0.17 ± 0.03	0.15 ± 0.02	0.665
myod1	**6.38 ± 0.42**	**9.19 ± 0.73**	**0.005**
myod2	2.97 ± 0.31	3.62 ± 0.46	0.162
myf5	0.36 ± 0.03	0.34 ± 0.03	0.833
myf6/mrf4	0.39 ± 0.02	0.40 ± 0.03	0.736
mstn	1.42 ± 0.34	2.36 ± 1.09	0.431
mef2a	12.55 ± 0.74	12.24 ± 0.63	0.757
mef2c	2.41 ± 0.11	2.45 ± 0.15	0.824
fst	0.95 ± 0.08	0.97 ± 0.11	0.877
il1β	0.09 ± 0.02	0.10 ± 0.01	0.695
il6	0.01 ± 0.00	0.01 ± 0.00	1.000
il8	0.03 ± 0.00	0.03 ± 0.00	0.611
il10	0.01 ± 0.00	0.01 ± 0.00	0.334
il12β	0.02 ± 0.00	0.02 ± 0.00	0.438
hif1α	1.49 ± 0.16	1.54 ± 0.18	0.806
pgc1α	0.21 ± 0.06	0.18 ± 0.07	0.717
pgc1β	0.35 ± 0.03	0.37 ± 0.06	0.766
cpt1a	1.61 ± 0.18	2.42 ± 0.14	0.734
cs	12.08 ± 0.80	11.54 ± 0.71	0.623
nd2	55.40 ± 4.80	51.19 ± 4.65	0.538
nd5	20.28 ± 2.13	19.08 ± 2.55	0.722
cox1	219.82 ± 23.09	232.48 ± 32.76	0.757

cox2	48.47 ± 5.20	50.27 ± 6.51	0.832
ucp3	7.32 ± 1.09	7.06 ± 1.31	0.876
sirt1	0.29 ± 0.02	0.29 ± 0.02	0.963
sirt2	0.72 ± 0.03	0.71 ± 0.05	0.823
cat	5.63 ± 0.27	5.20 ± 0.64	0.545
gpx4	0.91 ± 0.17	0.88 ± 0.30	0.930
gr	0.32 ± 0.03	0.30 ± 0.04	0.688
prdx3	2.65 ± 0.48	2.43 ± 0.22	0.676
prdx5	16.45 ± 1.05	15.17 ± 1.41	0.481
mn-sod/sod2	2.94 ± 0.13	2.77 ± 0.17	0.460
grp170	0.50 ± 0.04	0.49 ± 0.05	0.953
grp94	1.30 ± 0.15	1.15 ± 0.17	0.514
grp75	1.88 ± 0.12	1.86 ± 0.16	0.911

[1] Student's t-test *p-value.*
